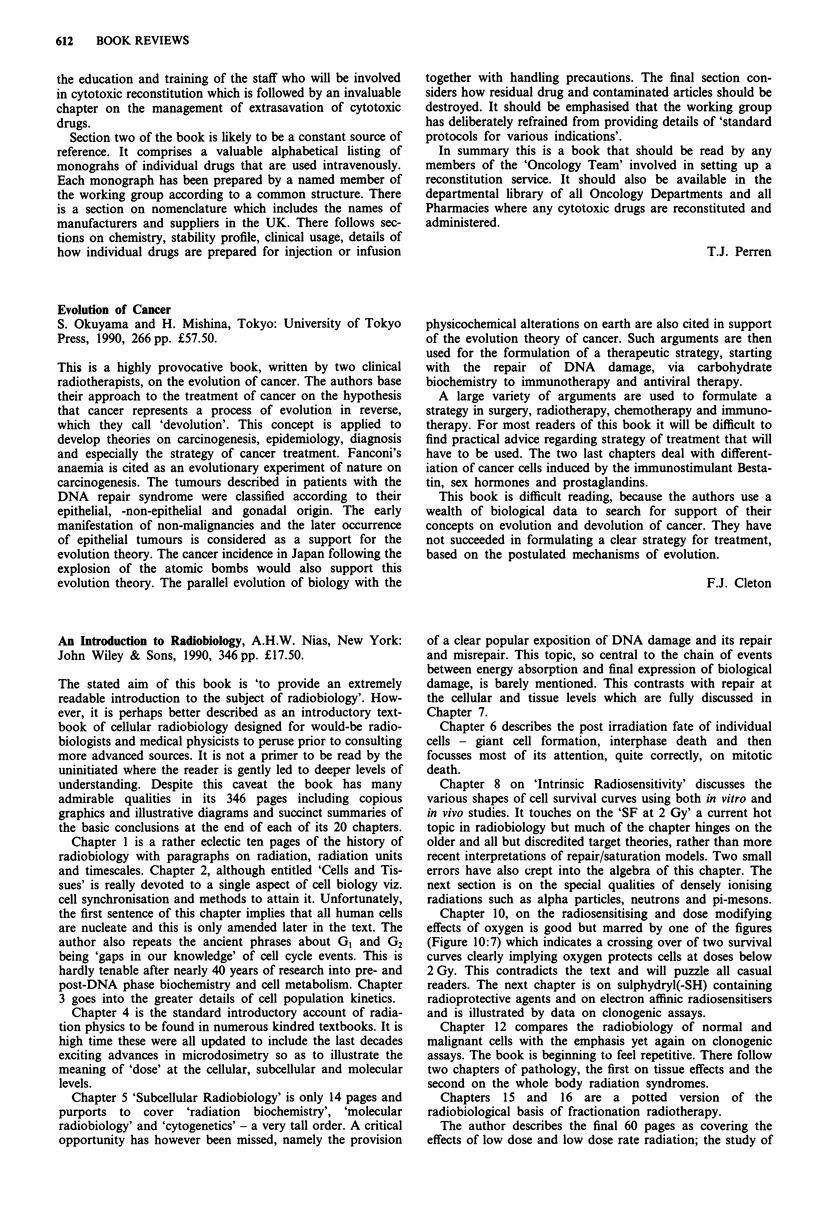# Evolution of Cancer

**Published:** 1991-09

**Authors:** F. J. Cleton


**Evolution of Cancer**
S. Okuyama and H. Mishina, Tokyo: University of Tokyo Press, 1990, 266 pp. £57.50.

This is a highly provocative book, written by two clinical radiotherapists, on the evolution of cancer. The authors base their approach to the treatment of cancer on the hypothesis that cancer represents a process of evolution in reverse, which they call 'devolution'. This concept is applied to develop theories on carcinogenesis, epidemiology, diagnosis and especially the strategy of cancer treatment. Fanconi's anaemia is cited as an evolutionary experiment of nature on carcinogenesis. The tumours described in patients with the DNA repair syndrome were classified according to their epithelial, -non-epithelial and gonadal origin. The early manifestation of non-malignancies and the later occurrence of epithelial tumours is considered as a support for the evolution theory. The cancer incidence in Japan following the explosion of the atomic bombs would also support this evolution theory. The parallel evolution of biology with the physicochemical alterations on earth are also cited in support of the evolution theory of cancer. Such arguments are then used for the formulation of a therapeutic strategy, starting with the repair of DNA damage, via carbohydrate biochemistry to immunotherapy and antiviral therapy.

A large variety of arguments are used to formulate a strategy in surgery, radiotherapy, chemotherapy and immunotherapy. For most readers of this book it will be difficult to find practical advice regarding strategy of treatment that will have to be used. The two last chapters deal with differentiation of cancer cells induced by the immunostimulant Bestatin, sex hormones and prostaglandins.

This book is difficult reading, because the authors use a wealth of biological data to search for support of their concepts on evolution and devolution of cancer. They have not succeeded in formulating a clear strategy for treatment, based on the postulated mechanisms of evolution.

F.J. Cleton